# Supplementary material for: Complex‐centric proteome profiling by SEC‐SWATH‐MS
Source: Mol Syst Biol. 2019 Jan 14;15(1):e8438. doi: 10.15252/msb.20188438 (PMC6346213; doi:10.15252/msb.20188438)
Supplement: Supplementary file 8 — Dataset EV7 [file MSB-15-e8438-s008.zip › feature_plots_string/Q9Y2A7.pdf]

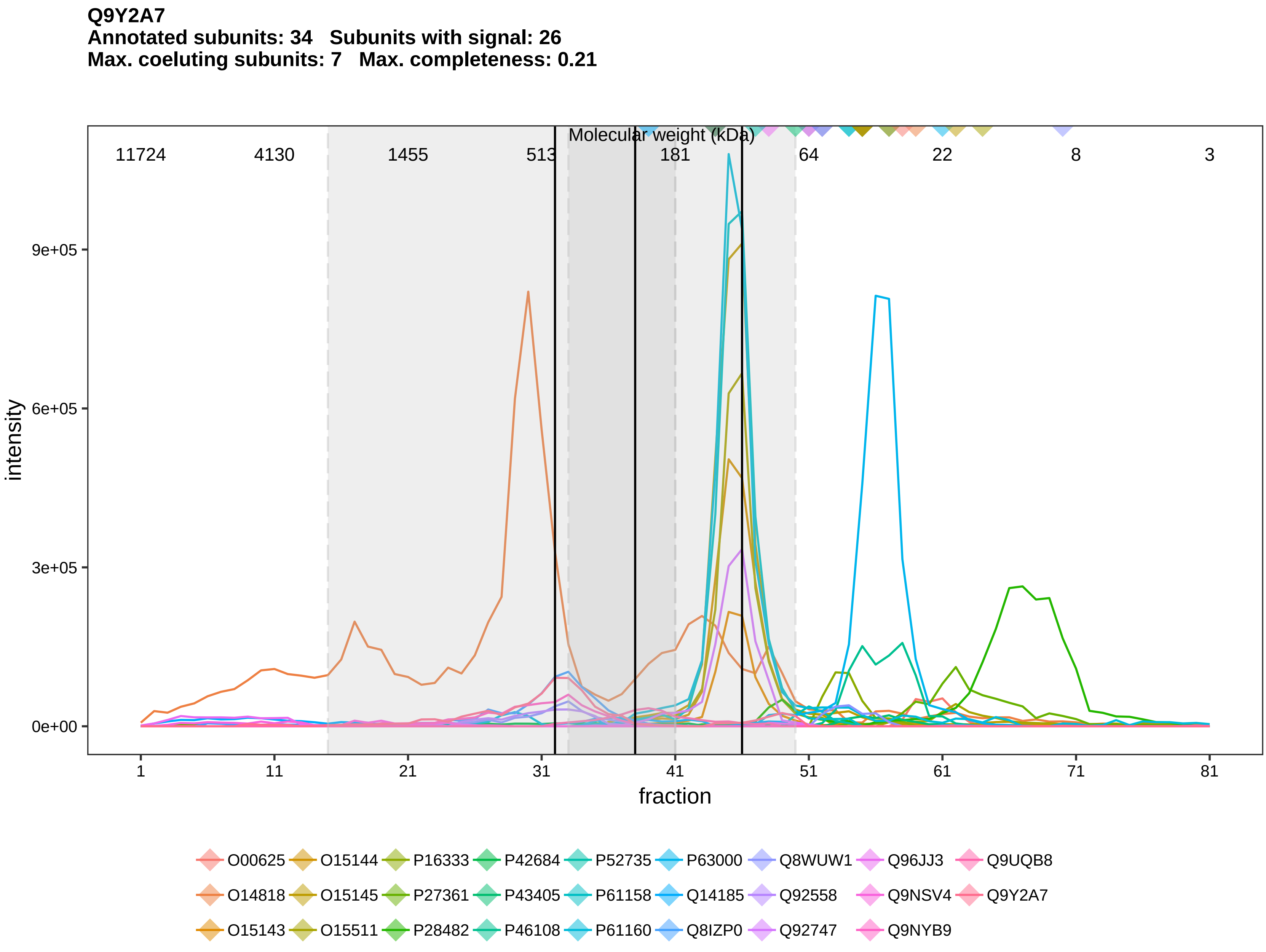

Q9Y2A7
Annotated subunits: 34 Subunits with signal: 26
Max. coeluting subunits: 7 Max. completeness: 0.21
Molecular weight (kDa)
11724
4130
1455
513
181
64
22
8
3
9e+05
6e+05
3e+05
0e+00
intensity
1
11
21
31
41
51
61
71
81
fraction
O00625
O15144
P16333
P42684
P52735
P63000
Q8WUW1
Q96JJ3
Q9UQB8
O14818
O15145
P27361
P43405
P61158
Q14185
Q92558
Q9NSV4
Q9Y2A7
O15143
O15511
P28482
P46108
P61160
Q8IZP0
Q92747
Q9NYB9
